# Is MRI better than CT for detecting a vascular component to dementia? A systematic review and meta-analysis

**DOI:** 10.1186/1471-2377-12-33

**Published:** 2012-06-06

**Authors:** Rebecca Beynon, Jonathan A C Sterne, Gordon Wilcock, Marcus Likeman, Roger M Harbord, Margaret Astin, Margaret Burke, Alysson Bessell, Yoav Ben-Shlomo, James Hawkins, William Hollingworth, Penny Whiting

**Affiliations:** 1 School of Social and Community Medicine, University of Bristol, Canynge Hall, 39 Whatley Road, Bristol, BS8 2PS, UK; 2 Frenchay Hospital, Beckspool Road, Bristol, BS16 1LE, UK; 3 Nuffield Department of Clinical Medicine, University of Oxford, John Radcliffe Hospital, Headley Way, Headington, Oxford, OX3 9DU, UK; 4 School of Oral and Dental Sciences, University of Bristol, Lower Maudlin Street, Bristol, BS1 2LY, UK

**Keywords:** Dementia, CT, MRI, Diagnosis, Systematic review

## Abstract

**Background:**

Identification of causes of dementia soon after symptom onset is important, because appropriate treatment of some causes of dementia can slow or halt its progression or enable symptomatic treatment where appropriate. The accuracy of MRI and CT, and whether MRI is superior to CT, in detecting a vascular component to dementia in autopsy confirmed and clinical cohorts of patients with VaD, combined AD and VaD (“mixed dementia”), and AD remain unclear. We conducted a systematic review and meta-analysis to investigate this question.

**Methods:**

We searched eight databases and screened reference lists to identify studies addressing the review question. We assessed study quality using QUADAS. We estimated summary diagnostic accuracy according to imaging finding, and ratios of diagnostic odds ratios (RDORs) for MRI versus CT and high versus low risk of bias.

**Results:**

We included 7 autopsy and 31 non-autopsy studies. There was little evidence that selective patient enrolment and risk of incorporation bias impacted on diagnostic accuracy (p = 0.12 to 0.95). The most widely reported imaging finding was white matter hyperintensities. For CT (11 studies) summary sensitivity and specificity were 71% (95% CI 53%-85%) and 55% (44%-66%). Corresponding figures for MRI (6 studies) were 95% (87%-98%) and 26% (12%-50%). General infarcts was the most specific imaging finding on MRI (96%; 95% CI 94%-97%) and CT (96%; 93%-98%). However, sensitivity was low for both MRI (53%; 36%-70%) and CT (52%; 22% to 80%). No imaging finding had consistently high sensitivity. Based on non-autopsy studies, MRI was more accurate than CT for six of seven imaging findings, but confidence intervals were wide.

**Conclusion:**

There is insufficient evidence to suggest that MRI is superior to CT with respect to identifying cerebrovascular changes in autopsy-confirmed and clinical cohorts of VaD, AD, and ‘mixed dementia’.

## Background

Dementia can be caused by different pathological processes that are often difficult to distinguish clinically, particularly in the early stages of the condition. Alzheimer’s disease (AD) is the most frequent, followed by vascular dementia (VaD), mixed pathology and dementia with Lewy Bodies [1]. Identification of causes of dementia soon after symptom onset is critical, because appropriate treatment of some causes of dementia can slow or halt its progression or enable symptomatic treatment where appropriate [2]. Acetylcholinesterase inhibitors may improve symptoms of Alzheimer’s disease, while dementia with a vascular pathology can be treated by addressing the vascular risk factors e.g. prescribing low dose aspirin or similar medication [3]. Failure to distinguish VaD from AD may lead to inappropriate treatment.

Autopsy is the reference standard for differential diagnosis of dementia in a research context. In clinical practice, and in research that does not follow patients until death, diagnostic criteria consisting of a combination of patient medical history, cognitive function assessment and imaging findings are often used. These include the National Institute of Neurological Disorders and Stroke and Association Internationale pour la Recherche et l’Ensignement en Neurosciences (NINDS-AIREN) criteria for VaD [4] and the National Institute of Neurological and Communicative Disorders and Stroke and the Alzheimer's Disease and Related Disorders Association (NINCDS-ADRDA) criteria for AD [5]. Neuroimaging is increasingly regarded as an essential part of the diagnostic work-up of a patient with dementia. Magnetic Resonance Imaging (MRI) has been advocated as the preferred imaging method in clinical guidelines [6], despite being more costly and (in some health systems) less readily available than computed tomography (CT).

Previously, neuroimaging was used to exclude abnormalities such as normal pressure hydrocephalus, tumours and subdural hematoma [7], but it is increasingly used to identify features consistent with the pathology of dementia subtypes such as cerebrovascular changes. The accuracy of MRI and CT, and whether MRI is superior to CT, in detecting a vascular component to dementia in clinical cohorts of patients with VaD, combined AD and VaD (“mixed dementia”), and AD remain unclear. We conducted a systematic review and meta-analysis to investigate this question.

## Methods

We produced a protocol for the review (available from the authors on request) detailing the proposed review methods.

### Literature search

We searched MEDLINE, EMBASE, BIOSIS, Science Citation Index, ZETOC, NTIS, Dissertation Abstracts, and the GrayLit networkfrom database inception to February 2011 for published and unpublished studies. We combined terms for each imaging test (Magnetic Resonance Imaging” OR “mri” OR “Computed Tomography” OR “ct scan$”) with terms for the target conditions (“Alzheimer Disease” OR “Vascular Dementia” OR “multi-infarct dementia”). We did not use a methodological search filter to identify diagnostic accuracy studies, because such filters may result in omission of relevant studies [8,9]. No language restrictions were applied.

### Study selection

Studies that assessed the accuracy of MRI and/or CT (index tests) for the detection of cerebrovascular changes in patients with VaD, AD or mixed dementia (target conditions) against an appropriate reference standard were eligible for inclusion. Eligible reference standards for VaD and AD included: autopsy; NINCDS-ADRDA [5] for AD; NINDS-AIREN [4] for VaD; Diagnostic and Statistical Manual of Mental Disorders (DSM) DSM-III [10], DSM-III R [11], DSM-IV [12]; State of California AD Diagnostic and Treatment Centre Criteria (ADDTC) [13]; and ICD-10 [14]. Any reported reference standard for mixed dementia was eligible. All subtypes of VaD (e.g. multi-infarct dementia; subcortical vascular ischemic dementia; and Binswanger’s dementia) were included in the VaD group. Studies had to report 2x2 performance data for one or more of the following cerebrovascular imaging findings: general infarcts; lacunar infarcts; non-lacunar infarcts; white matter hyperintensities (WMH); periventricular hyperintensities (PVH); basal ganglia hyperintensities (BGH); or a ‘global assessment’ finding, such as the presence of two or more findings. Two reviewers independently screened titles and abstracts. Full papers were assessed by one reviewer and checked by another; disagreements were resolved through consensus or referral to the review team.

### Data extraction and quality assessment

Data extraction and quality assessment were completed by one reviewer and checked by a second; disagreements were resolved through discussion or referral to a third reviewer. We extracted data on: inclusion/exclusion criteria, included patients, CT and MRI technical and operator details, reference standard, imaging finding, definition of a positive imaging finding, numbers of patients in each patient group (VaD, mixed dementia,AD or other diagnosis), and number of patients with positive imaging findings in each group. The patient groups were dichotomised as VaD or mixed dementia compared to AD or other diagnoses. This allowed construction of 2x2 tables of test performance, separately for each imaging finding assessed. Study quality was assessed using the Cochrane Collaboration’s adaption of the QUADAS tool [15].

### Statistical analyses

We calculated sensitivity, specificity, and the diagnostic odds ratio (DOR) of MRI and CT for the detection of VaD or Mixed dementia, for each 2x2 table. We plotted estimates of sensitivity and specificity from individual studies in summary receiver operating characteristic (SROC) space, separately for each imaging finding. We conducted separate analyses for studies that did and did not use autopsy as the reference standard. Summary sensitivity and specificity were estimated using the bivariate/HSROC meta-analysis models when sufficiently many studies (usually at least four) reported on the same imaging finding [16]. If too few studies were available to permit use of these models (for example, because the estimation procedure did not converge), univariate random-effects meta-analysis was carried out. We investigated the utility of different MRI and CT imaging findings to rule in or rule out a diagnosis of VaD or mixed dementia, by deriving positive and negative likelihood ratios from summary estimates of sensitivity and specificity. We used standard random-effects meta-analysis [17] to estimate summary DORs for each imaging finding, separately for MRI and CT, and then used meta-regression to calculate ratios of DORs (RDORs) comparing MRI with CT. We also estimated RDORs comparing MRI and CT in studies that reported direct comparisons of the two techniques. Estimates of the between-study variance τ^2^ were used to quantify heterogeneity. There were insufficient included studies to allow assessment of reporting bias.

We assessed the impact of patient spectrum (QUADAS item 1) and incorporation bias (QUADAS item 6) on diagnostic accuracy using meta-regression to calculate ratios of RDORs comparing the DOR in studies that were rated “no” or “unclear” with those rated “yes” on these QUADAS items, separately for MRI and CT. In these analyses, we selected one set of 2x2 data from each study on the basis of the following hierarchy: (1) global assessment, (2) white matter hyperintensities, (3) lacunar infarcts, (4) periventricular hyperintensities, (5) any other imaging finding. All analyses were done using Stata™ version 11, using the *metanmetandi* and *metareg* commands [18-20].

## Results

**Figure 1:**
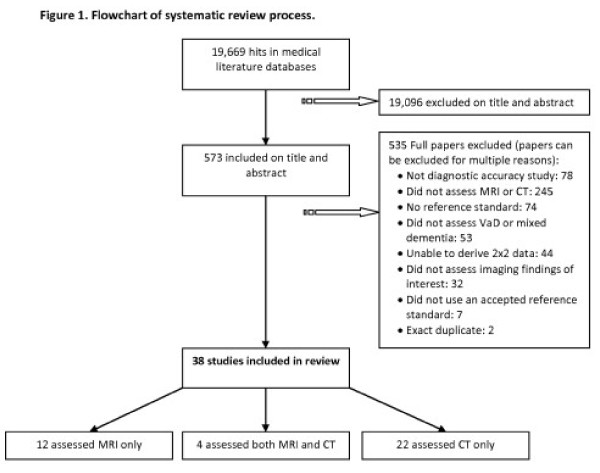
Flowchart of systematic review process.

The searches identified 19,669 titles and abstracts; 38 studies (4377 patients, range 23 to 683) were included in the review (Figure 1). Twenty-six studies (37 sets of 2x2 data) assessed CT, 16 (33 sets of 2x2 data) assessed MRI; 4 evaluated both CT and MRI and thus provided direct comparisons between the two techniques. Twenty studies were prospective cohorts, 6 were retrospective cohorts and 12 were case–control studies Table 1. Publication dates ranged from 1986 to 2010.

**Table 1:** Number of studies assessing each imaging method, according to study design and reference standard

	MRI only	CT only	MRI and CT	Total
Study Design				
Prospective cohort	4	14	2	20
Retrospective cohort	1	4	1	6
Case-control	7	4	1	12
Reference standard				
Autopsy	1	6	0	7
Non-autopsy	11	16	4	31
Total	12	22	4	38

Seven studies used autopsy as reference standard; all others used clinical criteria with or without imaging findings. VAD was confirmed by NINDS-AIREN (13 studies), DSM-III or DSM-III-R (16), and ICD10 (1). Reference standards used to define AD were NINCDS-ADRA (24 studies), DSM-III or DSM-III-R (6) and ICD10 (1). Six studies included mixed dementia patients, 2 used DSM-III-R, 2 used ADDTC, 1 ICD10, 1 Hachinski Ischemic Score and 1 history and examination as reference standard. Mean age, where reported, ranged from 66 years to 85 years and was generally higher in autopsy than non-autopsy studies. Individual study demographics and results are shown in the Additional file 1 .

**Figure 2:**
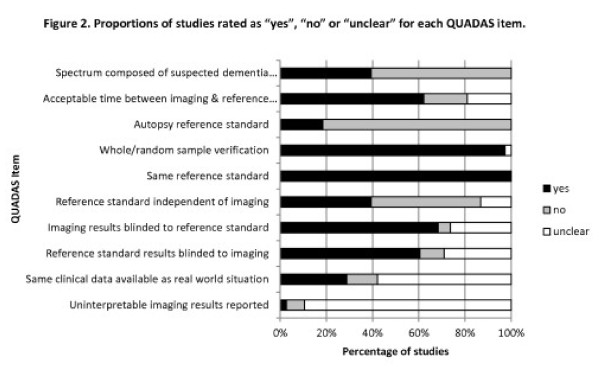
Proportions of studies rated as ’yes”, “no” or “unclear” for each QUADAS item.

The main limitations of the included studies were the potential for biased selection of patients and incorporation bias. Most studies (61%) did not enrol an appropriate patient spectrum, defined as patients with suspected dementia in whom the diagnosis had not been confirmed. There was a risk of incorporation bias in 23 (61%) of the non-autopsy studies, because the reference standard included the imaging findings. Other QUADAS items were classified as adequate or unclear in the majority of studies (Figure 2).

### Overall findings

**Figure 3:**
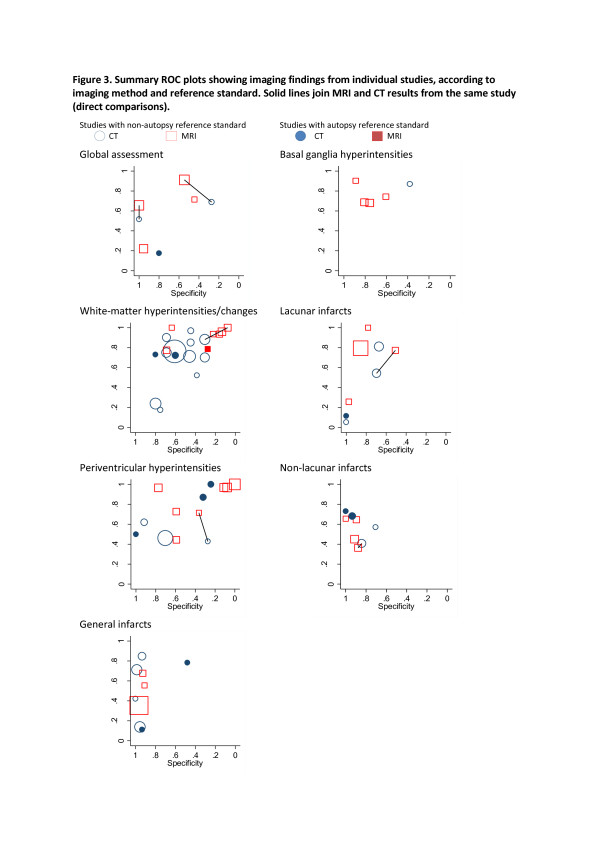
**Summary ROC plots showing imaging findings from individual studies, according to imaging method and reference standard.** Solid lines join MRI and CT results from the same study (direct comparisons).

There was substantial variation in estimates of accuracy reported in individual studies (Additional file 1). Figure 3 shows individual study estimates plotted in SROC space, separately for each imaging finding with different symbols according to imaging method (MRI or CT) and reference standard (autopsy or non-autopsy). These figures suggest that autopsy studies produced more of the outlying studies than the non-autopsy studies although there was no clear association with either sensitivity or specificity. Data from both direct and indirect comparisons suggested that MRI was more specific than CT with variable effects on sensitivity. The most specific imaging finding on both MRI and CT was general infarcts, but sensitivity was very heterogeneous for this finding. Non-lacunar infarcts also showed reasonable specificity, with heterogenous sensitivity. None of the findings had consistently high sensitivity. The most sensitive imaging finding appeared to be basal ganglia hyperintensities, but specificity was more variable and this finding was only assessed in five studies. White matter hyperintensities was the most commonly assessed finding, but results were heterogeneous.

### Autopsy studies

Six autopsy studies assessed CT and one assessed MRI (Table 2). White matter hyperintensities was the only imaging finding assessed on both MRI and CT; none of the studies reported a direct comparison between the two techniques. Based on three studies assessing white matter hyperintensities, the RDOR comparing CT (n = 2) with MRI (n = 1) was 0.28 (95% CI 0 to 55849), p = 0.42.

**Table 2:** Summary estimates of diagnostic accuracy from autopsy studies, according to imaging finding and method

Imaging finding	Number of studies	Sample size	Sensitivity (95% CI)	tau^2^ for sensitivity	Specificity (95% CI)	tau^2^ for specificity	Positive likelihood ratio (95% CI)	Negative likelihood ratio (95% CI)	DOR (95% CI)
CT									
White matter hyperintensities	2	84	73 (58, 84)	0	63 (46, 77)	0	1.96 (1.23, 3.12)	0.43 (0.25, 0.75)	4.5 (1.70, 12)
Global assessment	1	32	18 (6, 41)	—	80 (55, 93)	—	0.88 (0.21, 3.73)	1.03 (0.74, 1.44)	0.9 (0.20, 4.50)
Periventricular hyperintensities	3	31	85 (43, 98)	1.95	50 (11, 89)	2.12	1.70 (0.58, 5.05)	0.30 (0.04, 2.18)	5.6 (0.32, 100)
Lacunar infarcts	1	96	12 (4, 29)	—	100 (57, 100)	—	1.40 (0.08, 23.74)	0.96 (0.73, 1.27)	∞ (0.14, ∞)
Non-lacunar infarcts	2	79	70 (56, 81)	0	93 (80, 99)	0	12.75 (3.28, 49.60)	0.31 (0.20,0.48)	41 (8.20, 196)
General infarcts	2	123	41 (5, 89)	2.8	78 (32, 96)	1.7	1.82 (0.22,15.20)	0.76 (0.25,2.30)	2.4 (0.10, 57)
MRI									
White matter hyperintensities	1	43	79 (52, 92)	—	28 (15, 46)	—	1.09 (0.76, 1.55)	0.78 (0.24, 2.49)	1.4 (0.33, 5.80)

### Non-autopsy studies

We compared the DORs in studies that incorporated imaging findings in the reference standard with those that did not, and in studies that enrolled a selected sample of patients with those did not (Table 3). Each study contributed one set of 2 × 2 data to these analyses, based on the hierarchy described earlier. There was weak evidence that the accuracy of CT was overestimated in studies in which incorporation bias was present (RDOR 3.97, 95% CI 0.68 to 23.2) (p = 0.12). There was little evidence for an association between incorporation bias and MRI (p = 0.88), or between biased selection of patients and CT (p = 0.95) or MRI (p = 0.21). Based on these findings, we did not exclude studies with limitations in patient selection or at risk of incorporation bias from subsequent analyses.

**Table 3:** Comparisons of diagnostic odds ratios according to presence or absence of incorporation and selection bias, for each imaging method

Design feature	Imaging method	Ratio of diagnostic odds ratios (95% CI)	p-value
Incorporation bias	CT	3.97 (0.68,23.2)	0.12
	MRI	0.90 (0.20,3.95)	0.88
Selection bias	CT	1.05 (0.20,5.55)	0.95
	MRI	0.53 (0.18,1.51)	0.21

Table 4 shows summary estimates of sensitivity, specificity and positive and negative likelihood ratios for each imaging method and finding. Neither the individual imaging findings, nor the global assessment criteria, were found to have consistently high sensitivity. The most widely reported imaging finding was white matter hyperintensities. For CT (11 studies) summary sensitivity and specificity were 71% (95% CI 53% to 85%) and 55% (95% CI 44% to 66%). Corresponding figures for MRI (6 studies) were 95% (95% CI 87% to 98%) and 26% (95% CI 12% to 50%). This finding therefore had limited utility in ruling in or ruling out a diagnosis of VaD or mixed dementia. General infarcts was the most specific imaging finding on both MRI (96% (95% CI 94% to 97%)) and CT (96% (95% CI 93% to 98%)) with little heterogeneity (tau2 = 0). Corresponding positive likelihood ratios were also relatively high (LR + 13.08 (95% CI 7.64 to 22.4) for MRI and 12.22 (95% CI 5.59 to 26.7) for CT). However, sensitivity was low and showed substantial heterogeneity for both MRI (53% (95% CI 36% to 70%); tau^2^ = 0.21) and CT (52% (95% CI 22% to 80%), tau^2^ = 1.66).

**Table 4:** Summary estimates of diagnostic accuracy from non-autopsy studies, according to imaging finding and method

Imaging finding	Number of studies	Sample size	Sensitivity (95% CI)	tau^2^ for sensitivity	Specificity (95% CI)	tau^2^ for specificity	Positive likelihood ratio (95% CI)	Negative likelihood ratio (95% CI)
CT								
Global assessment	2	107	62 (50,72)	0.01	94 (1,100)	16.45	9.61 (0.01, 6410)	0.41 (0.24, 0.69)
White matter hyperintensities	11	196	71 (53,85)	1.53	55 (44,66)	0.48	1.58 (1.26, 1.98)	0.52 (0.32, 0.85)
Periventricular hyperintensities	3	354	49 (39,59)	0	69 (33,91)	1.48	1.57 (0.55, 4.44)	0.74 (0.45, 1.24)
Lacunar infarcts	3	667	42 (9,85)	2.93	86 (43,98)	2.74	3.22 (0.34, 30.9)	0.66 (0.27, 1.66)
Non-lacunar infarcts	2	1900	45 (34,56)	0	80 (68,88)	0.03	2.23 (1.34, 3.70)	0.69 (0.55, 0.87)
General infarcts	4	381	52 (22,80)	1.66	96 (93,98)	0	12.22 (5.59, 26.7)	0.50 (0.25, 1.01)
Basal ganglia hyperintensities	1	163	86 (78,92)	-	38 (28,49)	-	1.32 (1.09, 1.60)	0.38 (0.20, 0.73)
MRI								
Global assessment	4	170	69 (36,90)	1.57	86 (45,98)	3.14	4.86 (1.10, 21.5)	0.36 (0.16, 0.80)
White matter hyperintensities	6	334	95 (87,98)	0.75	26 (12,50)	1.41	1.29 (1.01, 1.66)	0.19 (0.07, 0.48)
Periventricular hyperintensities	7	315	92 (70,98)	3.61	24 (6,60)	4.45	1.19 (0.88, 1.63)	0.33 (0.10, 1.10)
Lacunar infarcts	4	599	75 (40,93)	1.82	83 (58,95)	1.45	4.49 (1.87, 10.8)	0.30 (0.11, 0.82)
Non-lacunar infarcts	4	514	53 (41,65)	0.13	91 (85,95)	0.05	6.11 (3.13, 11.9)	0.52 (0.39, 0.68)
General infarcts	3	955	53 (36,70)	0.21	96 (94,97)	0	13.08 (7.64, 22.4)	0.49 (0.35, 0.71)
Basal ganglia hyperintensities	4	396	77 (66,85)	0.10	78 (67,86)	0.17	3.46 (2.14, 5.60)	0.29 (0.18, 0.48)

MRI was found to have greater accuracy than CT for six of the seven imaging findings assessed (Table 5) with RDORs ranging from 1.78 (95% CI 0.11, 28.2) for periventricular hyperintensities to 2.68 (95% CI 0.33 to 22.0) for lacunar infarcts. However, confidence intervals were wide and evidence for an association was weak (p-values ranged from 0.15 to 0.64). The four studies that reported direct comparisons of MRI and CT supported the results from the indirect comparisons. However, RDORs were smaller for most imaging findings (range 1.12 to 1.86) with the exception of one study of global assessment (RDOR 14.81, 95% CI 1.73 to 127.14) and one of periventricular hyperintensities (RDOR 5.08, 95% CI 0.46 to 55.70).

**Table 5:** Summary estimates of diagnostic odds ratios from non-autopsy studies, according to imaging finding and method, and comparison of the diagnostic accuracy of the two methods

Imaging finding	MRI		CT		Comparison of MRI with CT		
	Number of studies	Diagnostic odds ratio (95% CI)	Number of studies	Diagnostic odds ratio (95% CI)	Ratio of diagnostic odds ratios (95% CI)	p-value	tau^2^
Global assessment	4	9.43 (0.67,132)	2	3.98 (0.10,160)	2.37 (0.03,222)	0.63	2.17
Lacunar infarcts	4	12.99 (3.02,55.9)	3	4.85 (1.06,22.2)	2.68 (0.33,22.0)	0.28	0.65
Non-lacunar infarcts	4	8.79 (3.20,24.2)	2	3.49 (1.27,9.59)	2.52 (0.60,10.5)	0.15	0
General infarcts	3	16.78 (2.01,139)	4	29.58 (3.99,219)	0.57 (0.03,10.5)	0.64	1.53
White matter hyperintensities	6	5.98 (1.90,18.9)	11	2.79 (1.43,5.44)	2.14 (0.57,8.09)	0.24	0.63
Periventricular hyperintensities	7	3.83 (0.71,20.6)	3	2.15 (0.24,19.3)	1.78 (0.11,28.2)	0.64	1.98
Basal ganglia hyperintensities	4	10.17 (1.62,63.9)	1	4.06 (0.15,109)	2.50 (0.58,108)	0.50	0.75

## Discussion

In this systematic review, we searched nearly 20,000 titles and abstracts in order to identify 38 studies that investigated the diagnostic accuracy of MRI or CT for detecting a vascular component to dementia. Only four of these studies assessed both imaging methods. Included studies were generally small and many were at high risk of bias due to the potential for biased selection of patients and possibility that test results were incorporated into the reference standard. However there was little evidence that these sources of bias impacted on estimates of accuracy. Only seven studies used autopsy as the reference standard, and their results were heterogeneous. Among the 31 studies that used a non-autopsy reference standard, no individual imaging finding was assessed in a majority of studies, and results were heterogeneous. White matter hyperintensities were the most frequently assessed imaging finding, but based on summary estimates of sensitivity and specificity this finding had limited utility for ruling in or ruling out a diagnosis of VaD or mixed dementia. The presence of general infarcts showed the greatest potential for ruling in a diagnosis of VaD or mixed dementia, but none of the findings appeared sufficiently sensitive to rule out a diagnosis of AD. Comparative analyses suggested that MRI may be more accurate than CT for distinguishing vascular or mixed dementia from Alzheimer’s disease and other conditions, but confidence intervals on estimated ratios of diagnostic odds ratios were wide.

We performed a comprehensive search, without language restrictions, to identify both published and unpublished literature: thus it is unlikely that relevant studies have been missed. We employed systematic review methods to minimise bias and errors during study selection, data extraction and quality assessment and used the most rigorous methods of meta-analysis for diagnostic accuracy data. We made both direct and indirect comparisons of the accuracy of CT and MRI, but were limited by the substantial between-study heterogeneity and small number of studies that directly compared the imaging methods. We assessed study quality using accepted criteria for diagnostic accuracy studies and investigated the effects of potential sources of bias in the analysis. Most of the included studies did not enrol an appropriate patient spectrum, which we defined as patients with symptoms of dementia in whom the diagnosis had not been confirmed. In practice, MRI and CT will have most clinical value if used at a relatively early stage in the diagnostic work-up of patients with symptoms of dementia, in order to help reach a definitive diagnosis and begin appropriate treatment early in the course of disease. Studies that did not assess MRI and/or CT in this context may produce less applicable, or biased, estimates of diagnostic accuracy: for example if they used a case–control design where cases already have a confirmed diagnosis of dementia subtype, or if they were conducted in patients with a longer duration of illness.

We stratified the analysis based on whether studies used an autopsy or non-autopsy reference standard. Because only a small number of autopsy studies were available, the impact of the type of reference standard on estimated diagnostic accuracy could not be evaluated with precision: no more than three autopsy studies assessed any individual imaging finding and most findings were assessed in only one or two studies. Although we considered autopsy to be the least biased reference standard there is a potential risk of disease progression bias as there will be a time lapse between the imaging and the autopsy examination. This means that some patients may not have had VaD orAD when they were assessed by MRI/CT but have developed one of these conditions before they died. This has the potential to impact on estimates of sensitivity and specificity, depending on whether the original reference standard is more likely to wrongly classify patients as VaD or AD. There was a risk of bias due to incorporation of test results in the reference standard, in many studies that used a non-autopsy reference standard. There was a suggestion that incorporation bias resulted in greater diagnostic accuracy for studies of CT but this was not found for studies of MRI. We would expect incorporation bias to increase agreement between the index test and reference standard leading to inflated estimates of sensitivity and specificity [21].

In the United States, the use of either CT or MRI as part of the diagnostic work-up of a dementia patient is recommended [22]. The UK National Institute for Health and Clinical Excellence (NICE) guidelines on dementia diagnosis state that structural imaging should be used in the assessment of suspected dementia to exclude other cerebral pathologies and to help establish the subtype diagnosis [6]. MRI is referred to as the preferred method to detect subcortical vascular changes, although it is acknowledged that CT could also be used. A 1988 narrative review by Joyce and Lishman [23], which discussed 9 studies, concluded that neither CT nor MRI are reliable in the differential diagnosis of AD and VaD.

Both CT and MRI technology have developed considerably in the time since the majority of the included studies were conducted. For example, helical CT with multiplanar reconstruction is now routinely used and has higher image resolution than the CT scans evaluated in the included studies. Modern CT may be considered to be preferential to MRI because it is quicker and much cheaper to buy and run, it is more comfortable for the patient and there are fewer contraindications to its use. It can be reconstructed in the coronal plane for direct visual assessment of hippocampal volume. These factors should be weighed against increased exposure to ionising radiation exposure with CT. In the future, fluorodeoxyglucose (FDG) - positron emission tomography (PET) may be useful in predicting decline in normal subjects and individuals with mild cognitive impairment [24]. Abeta-PET appears most useful in distinguishing AD from other dementias, although it has recently been suggested that a combination of Abeta- and FDG-PET may be more accurate. However, neither of these techniques is widely available in many hospital settings [25].

New diagnostic accuracy studies are needed to compare the utility of the latest generation of MRI and CT techniques in detecting a vascular component to dementia. The design of studies should aim to avoid the weaknesses of the studies located for this review. They should assess both MRI and CT in the same group of patients with symptoms of early dementia. Study size should be large enough to allow precise estimates of relative diagnostic accuracy. The reference standard should consist of accepted diagnostic criteria, without incorporating imaging findings, ideally supplemented by autopsy confirmation. Global assessment criteria for MRI and CT, based on the most useful individual imaging findings that are indicative of a vascular component to dementias, should be established, and their diagnostic accuracy quantified.

## Conclusions

This comprehensive, systematic literature review has shown that, despite its longstanding and widespread use, there is no strong evidence to suggest that MRI is more accurate than CT in identifying cerebrovascular changes in autopsy-confirmed and clinical cohorts of VaD, AD, and ‘mixed dementia’. There is a need for new, large, high quality studies comparing state of the art CT with MRI in patients with symptoms of early dementia.

## Competing interest

The authors declare that they have no competing interest.

## Authors’ contributions

GW, PW, JS, ML, YBS, WH developed the idea for the review and designed the review methods. JS, PW, RB, and RH analysed the data. RB, PW, JS, GW, ML, WH, MA, MW, RH, AB, YBS contributed to acquisition of data and/or interpretation of data. RB, PW, and JS drafted the manuscript. GW, ML, and WH critically revised the manuscript for important intellectual content. All authors read and approved the final manuscript.

## Funding

The review was funded by the United Kingdom Medical Research Council (Grant Code G0801405). GW was partly funded by the NIHR Biomedical Research Centre Programme, Oxford.

## Pre-publication history

The pre-publication history for this paper can be accessed here:

http://www.biomedcentral.com/1471-2377/12/33/prepub

## Supplementary Material

Additional file 1: Web Appendix: Included study details [26-64]. Click here for file

## References

[B1] Alzheimer’s Association 2011 Alzheimer’s Disease Facts and Figures 2011 http://www.alz.org/downloads/Facts_Figures_2011.pdf 10.1016/j.jalz.2011.02.004 21414557

[B2] Gifford DR Holloway RG Vickrey BG Systematic review of clinical prediction rules for neuroimaging in the evaluation of dementia Arch Inter Med 2000 160 2855 2862 10.1001/archinte.160.18.2855 11025796

[B3] Kirshner HS Vascular dementia: a review of recent evidence for prevention and treatment Curr Neurol Neurosci Rep 2009 9 437 442 10.1007/s11910-009-0065-y 19818230

[B4] Roman GC Tatemichi TK Erkinjuntti T Cummings JL Masdeu JC Garcia JH Vascular dementia: diagnostic criteria for research studies. Report of the NINDS-AIREN International Workshop Neurology 1993 43 250 260 10.1212/WNL.43.2.250 8094895

[B5] McKhann G Drachman D Folstein M Clinical diagnosis of Alzheimer’s disease: Report of the NINCDS-ADRDA Work Group under the auspices of the Department of Health and Human Services Task Force on Alzheimer’s disease Neurology 1984 34 939 944 10.1212/WNL.34.7.939 6610841

[B6] National Institute for Clinical Excellence Dementia: Supporting people with dementia and their carers in health and social care 2011 National Insititute for Clinical Excellence, London

[B7] Geldmacher DS Whitehouse PJ Evaluation of dementia New Eng J Med 1996 335 330 336 10.1056/NEJM199608013350507 8663868

[B8] Whiting P Westwood M Beynon R Burke M Sterne JA Glanville J Inclusion of methodological filters in searches for diagnostic test accuracy studies misses relevant studies J Clin Epidemiol 2010 64 6 602 607 21075596 10.1016/j.jclinepi.2010.07.006

[B9] Leeflang MM Scholten RJ Rutjes AW Reitsma JB Bossuyt PM Use of methodological search filters to identify diagnostic accuracy studies can lead to the omission of relevant studies J Clin Epidemiol 2006 59 234 240 10.1016/j.jclinepi.2005.07.014 16488353

[B10] American Psychiatric Association Diagnostic and Statistical Manual of Mental Disorders, Third Edition (DSM-III) 1980 American Psychiatric Association, Washington, DC

[B11] American Psychiatric Association Diagnostic and Statistical Manual of Mental Disorders, Third Edition (DSM-III-R) 1987 American Psychiatric Association, Washington, DC

[B12] American Psychiatric Association Diagnostic and Statistical Manual of Mental Disorders, Fourth Edition (DSM-IV) 1994 American Psychiatric Association, Washington, DC

[B13] Chui HC Victoroff JI Margolin D Jagust W Shankle R Katzmann R Criteria for the diagnosis of ischemic vascular dementia proposed by the state of California Alzheimer’s Disease Diagnostic and Treatment Centers Neurology 1992 42 473 480 10.1212/WNL.42.3.473 1549205

[B14] World Health Organisation The ICD-10 Classification of Mental and Behavioural Disorders: Clinical Descriptions and Diagnostic Guidelines 1992 World Health Organisation, Geneva, Switzerland

[B15] Whiting P Rutjes AW Reitsma JB Bossuyt PM Kleijnen J The development of QUADAS: a tool for the quality assessment of studies of diagnostic accuracy included in systematic reviews BMC Med Res Methodol 2003 3 25 10.1186/1471-2288-3-25 14606960 PMC305345

[B16] Harbord RM Deeks JJ Egger M Whiting P Sterne JA A unification of models for meta-analysis of diagnostic accuracy studies Biostatistics 2007 8 239 251 16698768 10.1093/biostatistics/kxl004

[B17] DerSimonian R Laird N Meta-analysis in clinical trials Control Clin Trials 1986 7 177 188 10.1016/0197-2456(86)90046-2 3802833

[B18] Harris RJ Deeks JJ Altman DG Bradburn MJ Harbord RM Sterne JAC metan: fixed- and random-effects meta-analysis Stata J 2008 8 3 28

[B19] Harbord RM Whiting P metandi: Meta-analysis of diagnostic accuracy using hierarchical logistic regression Stata J 2008 9 211 229

[B20] Harbord RM Higgins JPT Meta-regression in Stata Stata J 2008 8 493 519

[B21] Whiting P Rutjes AW Reitsma JB Glas AS Bossuyt PM Kleijnen J Sources of variation and bias in studies of diagnostic accuracy: a systematic review Ann Intern Med 2004 140 189 202 14757617 10.7326/0003-4819-140-3-200402030-00010

[B22] American Medical Directors Association Dementia in the long-term care setting. 48 2009 American Medical Directors Association (AMDA), Columbia

[B23] Joyce EM Lishman WA White matter changes in dementia Curr Opin Psychiatry 1988 1 475 479 10.1097/00001504-198807000-00014

[B24] Landau SM Harvey D Madison CM Reiman EM Foster NL Aisen PS Comparing predictors of conversion and decline in mild cognitive impairment Neurology 2010 75 230 238 10.1212/WNL.0b013e3181e8e8b8 20592257 PMC2906178

[B25] Mosconi L McHugh PF FDG- and amyloid-PET in Alzheimer’s disease: is the whole greater than the sum of the parts? Q J Nucl Med Mol Imaging 2011 55 250 264 21532539 PMC3290913

[B26] Barclay LL Linden C Murtagh R Medial temporal atrophy as a magnetic resonance imaging marker for Alzheimer’s disease J Neuroimaging 1992 2 3 131 135 10171577 10.1111/jon199223131

[B27] Del ST Hachinski V Merskey H Munoz DG Alzheimer’s disease with and without cerebral infarcts J Neurol Sci 2005 231 1–2 3 11 15792814 10.1016/j.jns.2004.08.016

[B28] Ettlin TM Staehelin HB Kischka U Ulrich J Scollo-Lavizzari G Wiggli U Computed tomography, electroencephalography, and clinical features in the differential diagnosis of senile dementia. A prospective clinicopathologic study Arch Neurol 1989 46 11 1217 1220 10.1001/archneur.1989.00520470081031 2818257

[B29] Erkinjuntti T Haltia M Palo J Sulkava R Paetau A Accuracy of the clinical diagnosis of vascular dementia: a prospective clinical and post-mortem neuropathological study J Neurol, Neurosurg Psychiatry 1988 51 8 1037 1044 10.1136/jnnp.51.8.1037 3216206 PMC1033111

[B30] Kondo N A study on the difference between clinical and neuropathological diagnoses of age-related dementing illnesses; correlations with Hachinski’s ischemic score Seishin Shinkeigaku Zasshi - Psychiatria et Neurologia Japonica 1995 97 10 825 846 8552726

[B31] Meguro K Matsushita M Yoshida R Otomo E Yamaguchi S Nakagawa T A clinicopathological study of senile dementia of Alzheimer’s type (SDAT) and white matter lesions of Binswanger’s type Jpn J Geriatr 1994 31 3 226 231 10.3143/geriatrics.31.226 8207874

[B32] Crum TA Luis CA Loewenstein DA Pascal S Bruce-Gregorius J Petito C MRI white matter hyperintensities in Alzheimer’s disease (AD) patients do not correlate with vascular disease: A clinico-pathological study Neurology 2003 60 65

[B33] Amar K Lewis T Wilcock G Scott M Bucks R The relationship between white matter low attenuation on brain CT and vascular risk factors: a memory clinic study Age & Ageing 1995 24 5 411 415 10.1093/ageing/24.5.411 8669345

[B34] Barber R Gholkar A Scheltens P Ballard C McKeith IG O’Brien JT Medial temporal lobe atrophy on MRI in dementia with Lewy bodies Neurology 1999 52 6 1153 1158 10.1212/WNL.52.6.1153 10214736

[B35] Charletta D Gorelick PB Dollear TJ Freels S Harris Y CT and MRI findings among African-Americans with Alzheimer’s disease, vascular dementia, and stroke without dementia Neurology 1995 45 8 1456 1461 10.1212/WNL.45.8.1456 7644040

[B36] Chen XS Application of ischemic score of Hachinski in differentiation of multi-infarct dementia Chung-Hua Shen Ching Ching Shen Ko Tsa Chih [Chinese Journal of Neurology & Psychiatry] 1992 25(6) 334 37 1304994

[B37] Engel PA Gelber J Does computed tomographic brain imaging have a place in the diagnosis of dementia? Arch Intern Med 1992 152 7 1437 1440 10.1001/archinte.1992.00400190067013 1627022

[B38] Erkinjuntti T Ketonen L Sulkava R Vuorialho M Palo J CT in the differential diagnosis between Alzheimer’s disease and vascular dementia Acta Neurol Scand 1987 75 4 262 270 10.1111/j.1600-0404.1987.tb07931.x 3591276

[B39] Erkinjuntti T Differential diagnosis between Alzheimer’s disease and vascular dementia: evaluation of common clinical methods Acta Neurol Scand 1987 76 6 433 442 10.1111/j.1600-0404.1987.tb03599.x 3434201

[B40] Erkinjuntti T Ketonen L Sulkava R Sipponen J Vuorialho M Iivanainen M Do white matter changes on MRI and CT differentiate vascular dementia from Alzheimer’s disease? J Neurol, Neurosurg Psychiatry 1987 50 1 37 42 10.1136/jnnp.50.1.37 3819754 PMC1033247

[B41] Frisoni GB Beltramello A Binetti G Bianchetti A Weiss C Scuratti A Computed tomography in the detection of the vascular component in dementia Gerontology 1995 41 2 121 128 7744267 10.1159/000213672

[B42] Hagiwara M A clinical study on the usefulness of CT and MRI imaging in evaluating differential diagnosis and the degree of dementia in vascular dementia Nippon Ika Daigaku Zasshi - J Nippon Med School 1990 57 3 265 275 10.1272/jnms1923.57.265 2376615

[B43] Kertesz A Polk M Carr T Cognition and white matter changes on magnetic resonance imaging in dementia Arch Neurol 1990 47 4 387 391 10.1001/archneur.1990.00530040029015 2322131

[B44] Nagga K Radberg C Marcusson J CT brain findings in clinical dementia investigation–underestimation of mixed dementia Dement Geriatr Cogn Disord 2004 18 1 59 66 10.1159/000077737 15084796

[B45] Purandare N Oude Voshaar RC McCollum C Jackson A Burns A Paradoxical embolisation and cerebral white matter lesions in dementia Br J Radiol 2008 81 961 30 34 17998278 10.1259/bjr/90498392

[B46] Scheltens P Kittner B Preliminary results from an MRI/CT-based database for vascular dementia and Alzheimer’s disease Ann NY Acad Sci 2000 903 542 546 10.1111/j.1749-6632.2000.tb06411.x 10818550

[B47] Schroder J Haan J Dickmann E Computerized tomography (CT) in multi-infarct (MID) and dementia of Alzheimer’s type (DAT) J Neural Transm - Parkinson’s Disease and Dementia Section 1989 1 1–2 127

[B48] Skoog I Palmertz B Andreasson LA The prevalence of white-matter lesions on computed tomography of the brain in demented and nondemented 85-year-olds. [Review] [53 refs] J Geriatr Psychiatry Neurol 1994 7 3 169 175 7916941 10.1177/089198879400700308

[B49] Staekenborg SS Koedam EL Henneman WJ Stokman P Barkhof F Scheltens P Progression of mild cognitive impairment to dementia: contribution of cerebrovascular disease compared with medial temporal lobe atrophy Stroke 2009 40 4 1269 1274 10.1161/STROKEAHA.108.531343 19228848

[B50] Steingart A Hachinski VC Lau C Fox AJ Fox H Lee D Cognitive and neurologic findings in demented patients with diffuse white matter lucencies on computed tomographic scan (leuko-araiosis) Arch Neurol 1987 44 1 36 39 10.1001/archneur.1987.00520130028013 3800720

[B51] Wahlund LO Basun H Almkvist O NderssonLundman G Julin P Saaf J White matter hyperintensities in dementia: does it matter? Magn Reson Imaging 1994 12 3 387 394 10.1016/0730-725X(94)92531-3 8007767

[B52] Wallin A Blennow K Uhlemann C Langstrom G Gottfries CG White matter low attenuation on computed tomography in Alzheimer’s disease and vascular dementia - Diagnostic and pathogenetic aspects Acta Neurol Scand 1989 80 6 518 523 10.1111/j.1600-0404.1989.tb03920.x 2618578

[B53] Zimny A Sasiadek M Leszek J Czarnecka A Trypka E Kiejna A Does perfusion CT enable differentiating Alzheimer’s disease from vascular dementia and mixed dementia? A preliminary report J Neurol Sci 2007 257 1–2 114 120 17362998 10.1016/j.jns.2007.01.051

[B54] Aharon-Peretz J Cummings JL Hill MA Vascular dementia and dementia of the Alzheimer type. Cognition, ventricular size, and leuko-araiosis Arch Neurol 1988 45 7 719 721 10.1001/archneur.1988.00520310025011 3260480

[B55] Butler RE Costa DC Greco A Ell PJ Katona CLE Differentiation between Alzheimer’s disease and multi-infarct dementia: SPECT vs MR imaging Int J Geriatr Psychiatry 1995 10 2 121 128 10.1002/gps.930100207

[B56] Du AT Schuff N Chao LL Kornak J Ezekiel F Jagust WJ White matter lesions are associated with cortical atrophy more than entorhinal and hippocampal atrophy Neurobiol Aging 2005 26 4 553 559 10.1016/j.neurobiolaging.2004.05.002 15653183

[B57] Ebmeier KP Besson JA Crawford JR Palin AN Gemmel HG Sharp PF Nuclear magnetic resonance imaging and single photon emission tomography with radio-iodine labelled compounds in the diagnosis of dementia Acta Psychiatr Scand 1987 75 5 549 556 10.1111/j.1600-0447.1987.tb02832.x 3496733

[B58] Endo R A study of the clinical and the neuroradiological findings in multi-infarct dementia and Alzheimer type dementia J Tokyo Women’s Med Coll 1989 59 6 693 704

[B59] Kobari M Meyer JS Ichijo M Leuko-araiosis, cerebral atrophy, and cerebral perfusion in normal aging Arch Neurol 1990 47 2 161 165 10.1001/archneur.1990.00530020061017 2302088

[B60] Kobari M Meyer JS Ichijo M Oravez WT Leukoaraiosis: correlation of MR and CT findings with blood flow, atrophy, and cognition Ajnr: Am J Neuroradiol 1990 11 2 273 281 2107711 PMC8334682

[B61] Lechner H Niederkorn K Schmidt R Does cerebrovascular insufficiency contribute to Alzheimer’s disease? Ann NY Acad Sci 1991 640 74 79 1776762 10.1111/j.1749-6632.1991.tb00194.x

[B62] London E de Leon MJ George AE Englund E Ferris S Gentes C Periventricular lucencies in the CT scans of aged and demented patients Biol Psychiatry 1986 21 10 960 962 10.1016/0006-3223(86)90270-2 3741912

[B63] Patankar TF Mitra D Varma A Snowden J Neary D Jackson A Dilatation of the Virchow-Robin space is a sensitive indicator of cerebral microvascular disease: study in elderly patients with dementia Ajnr: Am J Neuroradiol 2005 26 6 1512 1520 15956523 PMC8149063

[B64] Schmidt R Comparison of magnetic resonance imaging in Alzheimer’s disease, vascular dementia and normal aging Eur Neurol 1992 32 3 164 169 10.1159/000116816 1592074

