# Supplementary material for: Is MRI better than CT for detecting a vascular component to dementia? A systematic review and meta-analysis
Source: BMC Neurol. 2012 Jun 6;12:33. doi: 10.1186/1471-2377-12-33 (PMC3403932; doi:10.1186/1471-2377-12-33)
Supplement: Additional file 1: — Web Appendix: Included study details [26-64]. [file 1471-2377-12-33-S1.doc]

**Web Appendix: Included study details**

**A. Autopsy Studies**

| **Author** | **Sample size** | **Mean age (years)** | **% female** | **Patient spectrum** | **Number of patients** | | | | **Imaging finding** | **Sensitivity (%)** | **Specificity (%)** |
| --- | --- | --- | --- | --- | --- | --- | --- | --- | --- | --- | --- |
|  |  |  |  |  | **AD** | **VaD** | **Mixed** | **Other** |  |  |  |
| ***Cohort studies*** | | | | | | | | | | | |
| Barclay (1992)^26^ | 53 | 74 | 27 | All patients known to be clinically demented and received ante-mortem CT followed and autopsied through a dementia clinic. AD, VaD, mixed dementia and other dementias | 15 | 14 | 8 | 16 | CT 2 | 68 | 91 |
| Del Ser (2005)^27^ | 48 | 79 | 67 | Consecutive Caucasian dementia patients diagnosed with AD or mixed dementia at autopsy | 30 | 0 | 18 | 0 | CT 3 | 11 | 93 |
| Ettlin (1989)^28^ | 32 | 83 | 59 | AD, MID and mixed dementia diagnosed at autopsy | 15 | 11 | 6 | 0 | CT 8 | 18 | 80 |
| Erkinjuntti (1988)^29^ | 31 | 79 | 58 | Consecutive dementia patients who died and received an autopsy | 5 | 23 | 3 | 0 | CT 1 | 12 | 100 |
|  |  |  |  |  |  |  |  |  | CT 2 | 73 | 100 |
|  |  |  |  |  |  |  |  |  | CT 4 | 73 | 80 |
|  |  |  |  |  |  |  |  |  | CT 5 | 50 | 100 |
|  |  |  |  |  |  |  |  |  | CT 4 | 72 | 60 |
| Kondo (1995)^30^ | 48 | 76 | 69 | Consecutive dementia patients who underwent autopsy | 25 | 19 | 4 | 0 | CT 3 | 78 | 48 |
|  |  |  |  |  |  |  |  |  | CT 5 | 87 | 32 |
| Meguro (1994)^31^ | 44 | 84 | 77 | Patients with a clinical and pathological diagnosis of AD and AD with Binswanger’s disease | 33 | 0 | 11 | 0 | CT 5 | 100 | 24 |
| ***Case-control studies*** | | | | | | | | | | | |
| Crum (2003)^32^ | 43 | NR | NR | Patients with confirmed AD and mixed dementia on autopsy | 29 | 0 | 14 | 0 | MRI 4 | 79 | 28 |

**b. Non-autopsy Studies**

| **Author** | **Sample size** | **Mean age (years)** | **% female** | **Patient spectrum** | **Reference standard** | | | **N** | | | | **Imaging finding** | **Sensitivity %** | **Specificity %** |
| --- | --- | --- | --- | --- | --- | --- | --- | --- | --- | --- | --- | --- | --- | --- |
|  |  |  |  |  | **AD** | **VaD** | **Mixed** | **AD** | **VaD** | **Mixed** | **Other** |  |  |  |
| ***Diagnostic cohort studies*** | | | | | | | | | | | | | | |
| Amar (1995)^33^ | 216 | 71 | 55 | Patients referred to a memory disorders clinic and healthy controls. AD, VaD, mixed dementia, DLB, senile dementia, PD, alcoholism and healthy controls | N-ADRDA | DSM-III R |  | 76 | 33 | 5 | 102 | CT 4 | 71 | 47 |
| Barber (1999)^34^ | 133 | 77 | 46 | Patients aged >60 years with DSM-IV dementia. Includes possible VaD, possible AD and possible DLB | N-ADRDA | N-AIREN |  | 28 | 25 | 0 | 80 | MRI 4 | 96 | 13 |
|  |  |  |  |  |  |  |  |  |  |  |  | MRI 5 | 100 | 0 |
|  |  |  |  |  |  |  |  |  |  |  |  | MRI 6 | 68 | 76 |
| Charletta (1995)^35^ | 141 | 72 | 64 | African-American. AD, VaD and stroke patients without dementia | N-ADRDA | N-AIREN |  | 48 | 59 | 0 | 34 | CT 1 | 54 | 70 |
|  |  |  |  |  |  |  |  |  |  |  |  | CT 2 | 41 | 84 |
|  |  |  |  |  |  |  |  |  |  |  |  | CT 4 | 88 | 30 |
|  |  |  |  |  |  |  |  |  |  |  |  | MRI 1 | 77 | 51 |
|  |  |  |  |  |  |  |  |  |  |  |  | MRI 2 | 36 | 88 |
|  |  |  |  |  |  |  |  |  |  |  |  | MRI 4 | 100 | 8 |
| Chen (1992)^36^ | 50 | NR | NR | All dementia cases at a single hospital. AD, VaD, mixed dementia and other dementias | DSM-III R | DSM-III R | DSM-III R | 23 | 14 | 5 | 8 | CT 1 | 5 | 100 |
|  |  |  |  |  |  |  |  |  |  |  |  | CT 3 | 42 | 100 |
| Engel (1992)^37^ | 119 | 73 | 63 | Patients diagnosed as demented according to DSM-III R. AD, VaD, mixed dementia, possible dementia and other dementia caused by: PD, hydrocephalus, tumours, stroke, atypical dementia, syphilis, mitrochondrial encephalomyopathy | N-ADRDA | DSM-III R | DSM-III R | 56 | 8 | 5 | 50 | CT 3 | 85 | 94 |
| Erkinjuntti (1987)^38;39^ | 193 | 73 | 67 | Consecutive patients admitted to neurology centres. Results presented on AD, multi-infarct dementia or probable vascular dementia who had CT | N-ADRDA | N-AIREN |  | 68 | 125 | 0 | 0 | CT 3 | 71 | 99 |
| Erkinjuntti (1987)^40^ | 51 | 67 | 57 | Consecutive patients admitted to neurology centres. Results presented on AD or VaD who received both MRI and CT | N-ADRDA | DSM-III |  | 22 | 29 | 0 | 0 | CT 8 | 52 | 100 |
|  |  |  |  |  |  |  |  |  |  |  |  | MRI 2 | 66 | 100 |
|  |  |  |  |  |  |  |  |  |  |  |  | MRI 4 | 100 | 64 |
|  |  |  |  |  |  |  |  |  |  |  |  | MRI 5 | 97 | 77 |
|  |  |  |  |  |  |  |  |  |  |  |  | MRI 8 | 66 | 100 |
| Frisoni (1995)^41^ | 94 | 77 | 63 | Consecutive patients with cognitive problems who fulfilled the DSM-III-R criteria for dementia. AD, VaD, mixed dementia and uncertain dementia patients | N-ADRDA | N-AIREN |  | 57 | 10 | 20 | 7 | CT 4 | 90 | 69 |
| Hagiwara (1990)^42^ | 56 | 78 | NR | AD, VaD and mixed dementia | DSM-III | DSM-III | HIS | 11 | 34 | 11 | 0 | CT 8 | 69 | 27 |
|  |  |  |  |  |  |  |  |  |  |  |  | MRI 8 | 91 | 55 |
| Kertesz (1990)^43^ | 53 | 73 | NR | Patients referred for initial dementia diagnosis and normal controls. AD and VaD included in analysis. | N-ADRDA | N-AIREN |  | 27 | 11 | 0 | 15 | MRI 5 | 73 | 59 |
| Nagga (2004)^44^ | 163 | 77 | 66 | Consecutive in and outpatients evaluated for dementia. AD, VaD, mixed dementia and mild cognitive dysfunction patients | ICD-10 | ICD-10 | ICD-10 | 67 | 71 | 13 | 12 | CT 1 | 81 | 67 |
|  |  |  |  |  |  |  |  |  |  |  |  | CT 4 | 24 | 80 |
|  |  |  |  |  |  |  |  |  |  |  |  | CT 6 | 87 | 38 |
| Purandare (2008)^45^ | 108 | 75 | 45 | AD and VaD patients | N-ADRDA | N-AIREN |  | 57 | 51 | 0 | 0 | MRI 2 | 45 | 91 |
| Scheltens (2000)^46^ | 683 | NR | NR | AD and VaD patients | N-ADRDA | N-AIREN |  | 389 | 294 | 0 | 0 | CT 4 | 76 | 61 |
| Schroder (1989)^47^ | 55 | NR | NR | Consecutive demented patients with suspected AD and MID | DSM-III | DSM-III |  | 34 | 21 | 0 | 0 | CT 2 | 57 | 71 |
| Skoog (1994)^48^ | 246 | 85 | 70 | Representative sample of 85 year olds in Gothenburg. AD, VaD, other dementias and healthy controls | N-ADRDA | DSM-III R |  | 36 | 57 | 0 | 153 | CT 4 | 70 | 33 |
| Staekenborg (2009)^49^ | 483 | 65 | 47 | Consecutively included patients who attended an outpatient memory clinic and who were subsequently diagnosed with AD, VaD or MCI or in whom clinical investigations showed no abnormalities | N-ADRDA | N-AIREN |  | 210 | 34 | 0 | 239 | MRI 1 | 79 | 86 |
|  |  |  |  |  |  |  |  |  |  |  |  | MRI 3 | 35 | 96 |
| Steingart (1987)^50^ | 127 | 72 | 59 | Possible dementia patients. AD, mixed dementia, depressive pseudodementia, alcoholic dementia, progressive nuclear palsy, CJD, Parkinson's and pseudobulbar patients Severe MID patients excluded. | DSM-III | DSM-III |  | 91 | 0 | 8 | 28 | CT 4 | 75 | 69 |
| Wahlund (1994)^51^ | 79 | 77 | 75 | Consecutive patients with dementia symptoms. AD, VaD and possible AD. Early onset dementia, non-dementia or unclassifiable dementia excluded from analysis. | N-ADRDA | N-AIREN |  | 23 | 31 | 0 | 25 | MRI 5 | 77 | 69 |
| Wallin (1989)^52^ | 67 | 73 | 67 | Consecutive dementia patients with early onset AD, late onset AD and VaD | N-ADRDA | DSM-III |  | 47 | 20 | 0 | 0 | CT 4 | 85 | 45 |
| Zimny (2007)^53^ | 41 | 68 | 59 | AD, VaD and mixed dementia who underwent CT | N-ADRDA | N-AIREN | History/ exam | 24 | 8 | 9 | 0 | CT 4 | 18 | 75 |
| ***Case-control studies*** | | | | | | | | | | | | | | |
| Aharon-Peretz (1988)^54^ | 49 | 70 | 4 | MID and AD patients | N-ADRDA | DSM-III |  | 18 | 31 | 0 | 0 | CT 4 | 97 | 44 |
| Butler (1995)^55^ | 23 | 80 | 55 | AD and MID patients | N-ADRDA | DSM-III R |  | 9 | 7 | 0 | 7 | MRI 8 | 71 | 44 |
| Du (2005)^56^ | 140 | 76 | 46 | AD and mixed dementia patients and cognitively normal controls | N-ADRDA |  | ADDTC | 50 | 0 | 13 | 77 | MRI 1 | 100 | 78 |
| Ebmeier (1987)^57^ | 40 | 68 | 64 | Patients with dementia and normal controls recruited. Excluded dementia caused by metabolic, toxic, endocrine and nutritional factors. Results presented on MID and AD patients. | DSM-III | DSM-III |  | 22 | 18 | 0 | 0 | MRI 3 | 56 | 91 |
|  |  |  |  |  |  |  |  |  |  |  |  | MRI 5 | 44 | 59 |
|  |  |  |  |  |  |  |  |  |  |  |  | MRI 8 | 22 | 95 |
| Endo (1989)^58^ | 36 | 68 | 35 | AD, MID and multi-infarction without dementia | N-ADRDA | DSM-III-R |  | 15 | 21 | 0 | 9 | CT 5 | 62 | 92 |
| Kobari (1990)^59^ | 73 | 72 | NR | Normal volunteers, AD and MID patients | N-ADRDA | DSM-III R |  | 13 | 23 | 0 | 37 | CT 4 | 52 | 38 |
| Kobari (1990)^60^ | 31 | 66 | 48 | Patients with AD, MID, chronic cerebral infarctions and intact cognition, and elderly volunteers who were neurologically normal included | N-ADRDA | DSM-III-R |  | 9 | 14 | 0 | 8 | CT 5 | 43 | 27 |
|  |  |  |  |  |  |  |  |  |  |  |  | MRI 5 | 71 | 36 |
| Lechner (1991)^61^ | 88 | 69 | 67 | AD, VaD and normal controls | N-ADRDA | N-AIREN |  | 38 | 31 | 0 | 19 | MRI 1 | 26 | 97 |
|  |  |  |  |  |  |  |  |  |  |  |  | MRI 2 | 65 | 89 |
|  |  |  |  |  |  |  |  |  |  |  |  | MRI 4 | 94 | 16 |
|  |  |  |  |  |  |  |  |  |  |  |  | MRI 5 | 97 | 8 |
|  |  |  |  |  |  |  |  |  |  |  |  | MRI 6 | 74 | 61 |
| London (1986)^62^ | 305 | NR | NR | AD, VaD and normal aged controls | DSM-III | DSM-III |  | 151 | 65 | 0 | 89 | CT 3 | 14 | 95 |
|  |  |  |  |  |  |  |  |  |  |  |  | CT 5 | 46 | 70 |
| Patankar (2005)^63^ | 99 | 66 | 52 | Consecutive ischemic VaD, AD or FTD and healthy controls. Equivocal IVD cases excluded | N-ADRDA | N-AIREN |  | 35 | 16 | 0 | 48 | MRI 6 | 69 | 83 |
| Schmidt (1992)^64^ | 76 | 68 | 66 | Probable AD, VaD and normal controls | N-ADRDA | N-AIREN |  | 27 | 31 | 0 | 18 | MRI 3 | 68 | 93 |
|  |  |  |  |  |  |  |  |  |  |  |  | MRI 4 | 94 | 22 |
|  |  |  |  |  |  |  |  |  |  |  |  | MRI 5 | 97 | 11 |
|  |  |  |  |  |  |  |  |  |  |  |  | MRI 6 | 90 | 89 |

NR=not reported; AD=Alzheimer’s disease; MID=multi-infarct dementia; VaD=vascular dementia; MCI=mild cognitive impairment; DLB=dementia with lewy bodies; PD=Parkinson’s dementia; IVD=ischemic vascular dementia; CT=computed tomography; MRI=magnetic resonance imaging

| **Imaging finding** | **Description** |
| --- | --- |
| 1 | Lacunar infarcts (also known as subcortical, deep or basal ganglia infarcts) |
| 2 | Non-lacunar infarcts (also known as cortical or cerebral infarcts) |
| 3 | General infarcts |
| 4 | White matter hyperintensities |
| 5 | Periventricular hyperintensities |
| 6 | Basal ganglia hyperintensities |
| 8 | Global imaging assessment |

**References**

(26) Barclay LL, Linden C, Murtagh R. Medial temporal atrophy as a magnetic resonance imaging marker for Alzheimer's disease. *Journal of Neuroimaging* 1992; 2(3):131-135.

(27) Del ST, Hachinski V, Merskey H, Munoz DG. Alzheimer's disease with and without cerebral infarcts. *Journal of the Neurological Sciences* 2005; 231(1-2):3-11.

(28) Ettlin TM, Staehelin HB, Kischka U, Ulrich J, Scollo-Lavizzari G, Wiggli U et al. Computed tomography, electroencephalography, and clinical features in the differential diagnosis of senile dementia. A prospective clinicopathologic study. *Archives of Neurology* 1989; 46(11):1217-1220.

(29) Erkinjuntti T, Haltia M, Palo J, Sulkava R, Paetau A. Accuracy of the clinical diagnosis of vascular dementia: a prospective clinical and post-mortem neuropathological study. *Journal of Neurology, Neurosurgery & Psychiatry* 1988; 51(8):1037-1044.

(30) Kondo N. A study on the difference between clinical and neuropathological diagnoses of age-related dementing illnesses; correlations with Hachinski's ischemic score. *Seishin Shinkeigaku Zasshi - Psychiatria et Neurologia Japonica* 1995; 97(10):825-846.

(31) Meguro K, Matsushita M, Yoshida R, Otomo E, Yamaguchi S, Nakagawa T et al. A clinicopathological study of senile dementia of Alzheimer's type (SDAT) and white matter lesions of Binswanger's type. *Japanese Journal of Geriatrics* 1994; 31(3):226-231.

(32) Crum TA, Luis CA, Loewenstein DA, Pascal S, Bruce-Gregorius J, Petito C et al. MRI white matter hyperintensities in Alzheimer's disease (AD) patients do not correlate with vascular disease: A clinico-pathological study. *Neurology* 2003; 60(5 Supplement 1).

(33) Amar K, Lewis T, Wilcock G, Scott M, Bucks R. The relationship between white matter low attenuation on brain CT and vascular risk factors: a memory clinic study. *Age & Ageing* 1995; 24(5):411-415.

(34) Barber R, Gholkar A, Scheltens P, Ballard C, McKeith IG, O'Brien JT. Medial temporal lobe atrophy on MRI in dementia with Lewy bodies. *Neurology* 1999; 52(6):1153-1158.

(35) Charletta D, Gorelick PB, Dollear TJ, Freels S, Harris Y. CT and MRI findings among African-Americans with Alzheimer's disease, vascular dementia, and stroke without dementia. *Neurology* 1995; 45(8):1456-1461.

(36) Chen XS. Application of ischemic score of Hachinski in differentiation of multi-infarct dementia. *Chung-Hua Shen Ching Ching Shen Ko Tsa Chih [Chinese Journal of Neurology & Psychiatry]* 1992; 25(6):334-337.

(37) Engel PA, Gelber J. Does computed tomographic brain imaging have a place in the diagnosis of dementia? *Archives of Internal Medicine* 1992; 152(7):1437-1440.

(38) Erkinjuntti T, Ketonen L, Sulkava R, Vuorialho M, Palo J. CT in the differential diagnosis between Alzheimer's disease and vascular dementia. *Acta Neurologica Scandinavica* 1987; 75(4):262-270.

(39) Erkinjuntti T. Differential diagnosis between Alzheimer's disease and vascular dementia: evaluation of common clinical methods. *Acta Neurologica Scandinavica* 1987; 76(6):433-442.

(40) Erkinjuntti T, Ketonen L, Sulkava R, Sipponen J, Vuorialho M, Iivanainen M. Do white matter changes on MRI and CT differentiate vascular dementia from Alzheimer's disease? *Journal of Neurology, Neurosurgery & Psychiatry* 1987; 50(1):37-42.

(41) Frisoni GB, Beltramello A, Binetti G, Bianchetti A, Weiss C, Scuratti A et al. Computed tomography in the detection of the vascular component in dementia. *Gerontology* 1995; 41(2):121-128.

(42) Hagiwara M. A clinical study on the usefulness of CT and MRI imaging in evaluating differential diagnosis and the degree of dementia in vascular dementia. *Nippon Ika Daigaku Zasshi - Journal of the Nippon Medical School* 1990; 57(3):265-275.

(43) Kertesz A, Polk M, Carr T. Cognition and white matter changes on magnetic resonance imaging in dementia. *Archives of Neurology* 1990; 47(4):387-391.

(44) Nagga K, Radberg C, Marcusson J. CT brain findings in clinical dementia investigation--underestimation of mixed dementia. *Dementia & Geriatric Cognitive Disorders* 2004; 18(1):59-66.

(45) Purandare N, Oude Voshaar RC, McCollum C, Jackson A, Burns A. Paradoxical embolisation and cerebral white matter lesions in dementia. *British Journal of Radiology* 2008; 81(961):30-34.

(46) Scheltens P, Kittner B. Preliminary results from an MRI/CT-based database for vascular dementia and Alzheimer's disease. *Annals of the New York Academy of Sciences* 2000; 903:542-546.

(47) Schroder J, Haan J, Dickmann E. Computerized tomography (CT) in multi-infarct (MID) and dementia of Alzheimer's type (DAT). *Journal of Neural Transmission - Parkinson's Disease and Dementia Section* 1989; 1(1-2):127.

(48) Skoog I, Palmertz B, Andreasson LA. The prevalence of white-matter lesions on computed tomography of the brain in demented and nondemented 85-year-olds. [Review] [53 refs]. *Journal of Geriatric Psychiatry & Neurology* 1994; 7(3):169-175.

(49) Staekenborg SS, Koedam EL, Henneman WJ, Stokman P, Barkhof F, Scheltens P et al. Progression of mild cognitive impairment to dementia: contribution of cerebrovascular disease compared with medial temporal lobe atrophy. *Stroke* 2009; 40(4):1269-1274.

(50) Steingart A, Hachinski VC, Lau C, Fox AJ, Fox H, Lee D et al. Cognitive and neurologic findings in demented patients with diffuse white matter lucencies on computed tomographic scan (leuko-araiosis). *Archives of Neurology* 1987; 44(1):36-39.

(51) Wahlund LO, Basun H, Almkvist O, ndersson-Lundman G, Julin P, Saaf J. White matter hyperintensities in dementia: does it matter? *Magnetic Resonance Imaging* 1994; 12(3):387-394.

(52) Wallin A, Blennow K, Uhlemann C, Langstrom G, Gottfries CG. White matter low attenuation on computed tomography in Alzheimer's disease and vascular dementia - Diagnostic and pathogenetic aspects. *Acta Neurologica Scandinavica* 1989; 80(6):518-523.

(53) Zimny A, Sasiadek M, Leszek J, Czarnecka A, Trypka E, Kiejna A. Does perfusion CT enable differentiating Alzheimer's disease from vascular dementia and mixed dementia? A preliminary report. *Journal of the Neurological Sciences* 2007; 257(1-2):114-120.

(54) Aharon-Peretz J, Cummings JL, Hill MA. Vascular dementia and dementia of the Alzheimer type. Cognition, ventricular size, and leuko-araiosis. *Archives of Neurology* 1988; 45(7):719-721.

(55) Butler RE, Costa DC, Greco A, Ell PJ, Katona CLE. Differentiation between Alzheimer's disease and multi-infarct dementia: SPECT vs MR imaging. *International Journal of Geriatric Psychiatry* 1995; 10(2):121-128.

(56) Du AT, Schuff N, Chao LL, Kornak J, Ezekiel F, Jagust WJ et al. White matter lesions are associated with cortical atrophy more than entorhinal and hippocampal atrophy. *Neurobiology of Aging* 2005; 26(4):553-559.

(57) Ebmeier KP, Besson JA, Crawford JR, Palin AN, Gemmel HG, Sharp PF et al. Nuclear magnetic resonance imaging and single photon emission tomography with radio-iodine labelled compounds in the diagnosis of dementia. *Acta Psychiatrica Scandinavica* 1987; 75(5):549-556.

(58) Endo R. A study of the clinical and the neuroradiological findings in multi-infarct dementia and Alzheimer type dementia. *Journal of Tokyo Women's Medical College* 1989; 59(6):693-704.

(59) Kobari M, Meyer JS, Ichijo M. Leuko-araiosis, cerebral atrophy, and cerebral perfusion in normal aging. *Archives of Neurology* 1990; 47(2):161-165.

(60) Kobari M, Meyer JS, Ichijo M, Oravez WT. Leukoaraiosis: correlation of MR and CT findings with blood flow, atrophy, and cognition. *Ajnr: American Journal of Neuroradiology* 1990; 11(2):273-281.

(61) Lechner H, Niederkorn K, Schmidt R. Does cerebrovascular insufficiency contribute to Alzheimer's disease? *Annals of the New York Academy of Sciences* 1991; 640:74-79.

(62) London E, de Leon MJ, George AE, Englund E, Ferris S, Gentes C et al. Periventricular lucencies in the CT scans of aged and demented patients. *Biological Psychiatry* 1986; 21(10):960-962.

(63) Patankar TF, Mitra D, Varma A, Snowden J, Neary D, Jackson A. Dilatation of the Virchow-Robin space is a sensitive indicator of cerebral microvascular disease: study in elderly patients with dementia. *Ajnr: American Journal of Neuroradiology* 2005; 26(6):1512-1520.

(64) Schmidt R. Comparison of magnetic resonance imaging in Alzheimer's disease, vascular dementia and normal aging. *European Neurology* 1992; 32(3):164-169.
